# Changing Epidemiology of Human Brucellosis, China, 1955–2014

**DOI:** 10.3201/eid2302.151710

**Published:** 2017-02

**Authors:** Shengjie Lai, Hang Zhou, Weiyi Xiong, Marius Gilbert, Zhuojie Huang, Jianxing Yu, Wenwu Yin, Liping Wang, Qiulan Chen, Yu Li, Di Mu, Lingjia Zeng, Xiang Ren, Mengjie Geng, Zike Zhang, Buyun Cui, Tiefeng Li, Dali Wang, Zhongjie Li, Nicola A. Wardrop, Andrew J. Tatem, Hongjie Yu

**Affiliations:** Division of Infectious Disease, Key Laboratory of Surveillance and Early-Warning on Infectious Disease, Chinese Center for Disease Control and Prevention, Beijing, China (S. Lai, H. Zhou, W. Xiong, Z. Huang, J. Yu, W. Yin, L. Wang, Q. Chen, Y. Li, D. Mu, L. Zeng, X. Ren, M. Geng, Z. Zhang, Z. Li, H. Yu);; WorldPop, Geography and Environment, University of Southampton, Southampton, UK (S. Lai, N.A. Wardrop, A.J. Tatem);; Flowminder Foundation, Stockholm, Sweden (S. Lai, A.J. Tatem);; School of Public Health, Fudan University, Key Laboratory of Public Health Safety, Ministry of Education, Shanghai, China (S. Lai, H. Yu);; Universite´ Libre de Bruxelles, Brussels, Belgium (M. Gilbert); Fonds National de la Recherche Scientifique, Brussels (M. Gilbert);; Institute of Pathogen Biology, Chinese Academy of Medical Sciences, Beijing (J. Yu);; Zhejiang University, Hangzhou, China (Z. Zhang);; National Institute for Communicable Disease Control and Prevention, Chinese Center for Disease Control and Prevention, Beijing (B. Cui);; Base of Plague and Brucellosis Prevention and Control, Chinese Center for Disease Control and Prevention, Beijing (T. Li, D. Wang)

**Keywords:** Brucellosis, humans, epidemiology, zoonoses, China, bacteria, Brucella

## Abstract

Brucellosis, a zoonotic disease, was made statutorily notifiable in China in 1955. We analyzed the incidence and spatial–temporal distribution of human brucellosis during 1955–2014 in China using notifiable surveillance data: aggregated data for 1955–2003 and individual case data for 2004–2014. A total of 513,034 brucellosis cases were recorded, of which 99.3% were reported in northern China during 1955–2014, and 69.1% (258, 462/374, 141) occurred during February–July in 1990–2014. Incidence remained high during 1955–1978 (interquartile range 0.42–1.0 cases/100,000 residents), then decreased dramatically in 1979–1994. However, brucellosis has reemerged since 1995 (interquartile range 0.11–0.23 in 1995–2003 and 1.48–2.89 in 2004–2014); the historical high occurred in 2014, and the affected area expanded from northern pastureland provinces to the adjacent grassland and agricultural areas, then to southern coastal and southwestern areas. Control strategies in China should be adjusted to account for these changes by adopting a One Health approach.

Brucellosis is a bacterial zoonosis caused by *Brucella* spp., which can be transmitted from animal reservoirs, such as cattle, sheep, goats, and pigs, to humans through direct contact with infected animals or ingestion of unpasteurized animal products (*1*–*3*). The global epidemiology of brucellosis has drastically changed over the past decades, particularly in industrialized countries where the disease was previously endemic but is now mainly associated with returning travelers. However, brucellosis remains a serious concern in low- and middle-income countries, which have most new human cases globally (estimated ≈500,000 cases annually) and major economic losses in animal production resulting from the adverse effects of infection on livestock reproduction (*2*,*4*,*5*). Additionally, human brucellosis is of particular concern because of high initial treatment failure, substantial residual disability of infected patients, and relapse rates (*1*,*6*). Moreover, *Brucella* spp. are highly infectious through the aerosol route, making them a potential agent of biological weapons and bioterrorism (*7*).

In China, brucellosis was first recorded as Malta fever for 2 foreigners in Shanghai in 1905, but several patients in China who had similar clinical symptoms had been observed in the 10 years before this report (*8*). After this report, 3 cases were reported from Chongqing in 1906 (*9*). The first person with a definite diagnosis of *Brucella* infection by serologic tests was reported from Fujian in 1916 (*10*). Subsequently, *Brucella* sp. was isolated from a foreigner and his goats who traveled from Punjab, India, to Henan Province, China, in 1925 (*11*), and human infection in a laboratory setting was reported in Beijing in 1936 (*12*). Thus, human brucellosis was seen in China before 1950, especially in the northern provinces (*13*).

Since 1950, activities for prevention and control of brucellosis have been gradually introduced in mainland China (*14*,*15*). During 1950–1963, the reporting for human brucellosis was established nationwide, and some surveys were conducted. Vaccination for animals and humans was implemented as the main control measure during 1964–1976 in regions with severe epidemics, such as Inner Mongolia, Xinjiang, Qinghai, Ningxia, and Henan Provinces (*14*). During 1977–1988, a national program for brucellosis control was conducted with the introduction of diagnostic criteria, treatment protocols, and control measures, and vaccination of domestic animals was used as the main control measure. National sentinel surveillance was established in 1990 to monitor the seroprevalence of brucellosis in humans and animals (*16*).

During the past decade, outbreaks of human brucellosis have been reported in increasing numbers and with an apparent geographic expansion from the historically affected north of China (*17*,*18*) to southern provinces where nonoccupational exposure might be more common because of the increasing movement of humans, animals, and animal food products from brucellosis-endemic regions (*19*–*21*). The epidemiology of human brucellosis clearly presented major challenges in China during the past 60 years, but studies reporting the spatial–temporal patterns of human brucellosis with high-quality, nationwide incidence data are lacking (*2*,*4*,*14*,*22*,*23*). We describe the magnitude and distribution of human brucellosis in mainland China using the notifiable reporting data for 1955–2014 and emphasize its recent reemergence. Improving our understanding of the changing epidemiology of brucellosis and identifying high-risk areas can help in formulating plans for national strategies to prevent and control brucellosis.

## Methods

### Data Source and Ethical Considerations

On July 5, 1955, human brucellosis was made statutorily notifiable in China: all probable or laboratory-confirmed new brucellosis cases were required to be reported (Technical Appendix Table 1). In this study, we used 2 datasets because the requirements for reporting changed during the study period (*24*). One comprises the number of brucellosis cases aggregated by case-patient sex, age group, and occupation; incidence rate; death rate; and case-fatality ratio (Technical Appendix Table 2), reported monthly through paper-based post or electronic files during 1955–2003. The other consists of individual brucellosis cases reported by doctors within 24 hours after diagnosis to the online National Notifiable Infectious Disease Reporting Information System at the Chinese Center for Disease Control and Prevention during 2004–2014. (Variables in individual datasets are available in Technical Appendix Table 3.) All data used in this study were anonymized so that individual patients could not be identified.

The National Health and Family Planning Commission of China determined that the collection of data from human cases of brucellosis was part of continuing public health surveillance of a notifiable infectious disease and was exempt from institutional review board assessment. All data were supplied and analyzed in an anonymous format, without access to personal identifying information.

### Case Definition

Brucellosis cases have been classified as probable (clinically diagnosed) or confirmed (laboratory confirmed) in accordance with the guidelines for human brucellosis diagnosis issued by the Chinese national health authorities in 1977, 1988, 1996, and 2007, which were successively used during 1977–2014 (Technical Appendix Table 4). Probable cases are diagnosed by local experienced physicians according to patient anamnesis, epidemiologic exposure, clinical manifestations, and/or positive results of presumptive laboratory tests, including the plate agglutination test and the intradermal allergic reaction test. Confirmed cases are probable cases with 1 positive result of the following tests: standard tube agglutination test, complement fixation test, Coombs test, cysteine test for serologic diagnosis, or positive *Brucella* spp. isolation (*1*).

### Data Analysis

Our analysis comprised all probable and confirmed cases in persons with illness onset from January 1, 1955, through December 31, 2014. According to the National Mid-term and Long-term Animal Disease Control Plan of China (*25*), all the provinces in northern China were identified as the key regions for brucellosis control. Therefore, we aggregated the surveillance data of each province to northern and southern China (Technical Appendix Table 5), as previously reported (*26*), to examine spatial–temporal patterns by region. To eliminate the potential effect of the introduction of Internet-based reporting on the increasing number of cases since 2004, we made a time-series prediction of the number of cases in 2004 on the basis of data for 1993–2003, using the Holt exponential smoothing method with a 95% CI (*27*). Then we compared the upper value of the 95% CI with the actual number of cases in 2004 to calculate the excess proportion of cases that might have contributed to improved data reporting. Adjusted incidence rates for 2004–2014 were estimated by using this excess proportion and plotted as an epidemic curve. We also predicted the monthly numbers of cases during the next 5 years (2015–2019) by Holt-Winters exponential smoothing on the basis of data reported during 2004–2014 to explore the trend of incidence with seasonality (*27*).

We created a heat map of the yearly incidence rate to visualize the long-term change over the 60-year period by province. We also created a heat map of the monthly number of cases reported during 2005–2014 by province, standardized by the yearly number in each province, and plotted a heat map of average weekly proportions of case numbers by province to explore the seasonal pattern during 2005–2014. To test the differences between northern and southern China, we used Mann-Whitney U tests with a significance level of α = 0.05 to test for differences in the time from illness onset to diagnosis and χ^2^ with a significance level of α = 0.05 to test the differences in the proportion of imported cases. The *R* statistical software (version 3.1.2, R Foundation for Statistical Computing, Vienna, Austria) with the package *forecast* (version 6.1), was used to produce the graphs and heat maps and to perform statistical analyses and prediction, and *ArcGIS* 10.2.2 (ESRI, Redlands, CA, USA) was used to plot the geographic patterns.

## Results

### Demographic Features

**Figure 1:**
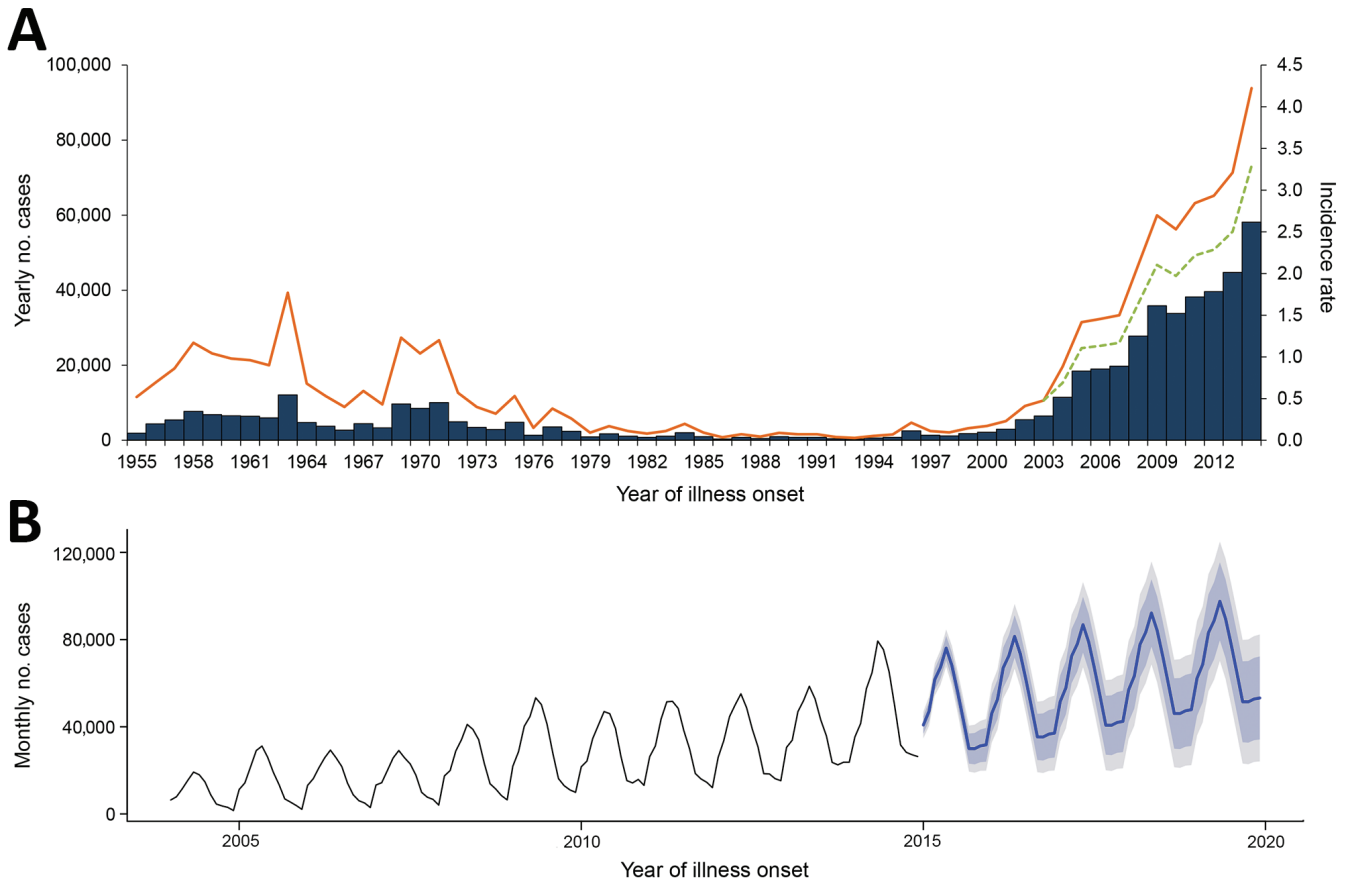
Reported human brucellosis cases (N = 513,034), mainland China, 1955–2014. A) Aggregated number of cases (blue bars) and annual incidence rate (orange line) per 100,000 residents reported by year. The adjusted incidence rate (green dashed line) was estimated by an excess proportion (22.06%) that might be attributed to the effect of Internet-based reporting since 2004 (see Methods). B) Forecast of the monthly number of cases (blue line) during 2015–2019 by Holt-Winters exponential smoothing with 80% CIs (light gray) and 95% CIs (dark gray) based on monthly numbers for 2004–2014.

**Figure 2:**
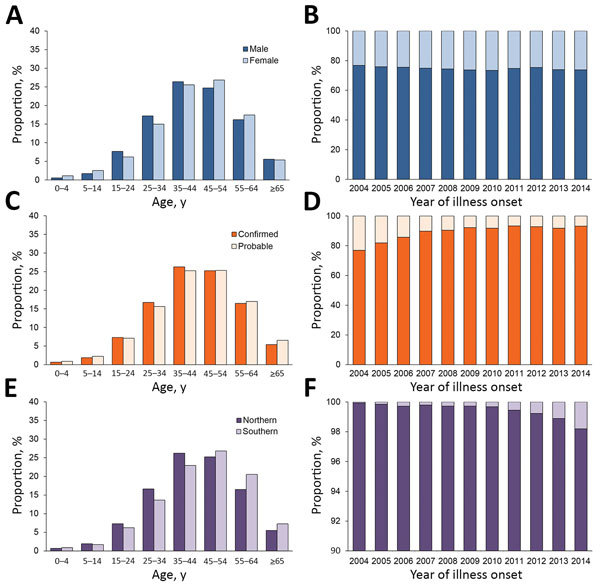
Age distribution and proportion of 346,682 human brucellosis patients, mainland China, 2004–2014. A) Age distribution by sex. B) Annual proportion of patients by sex. C) Age distribution of persons with probable and confirmed cases. D) Annual proportion of probable and confirmed cases. E) Age distribution of patients in northern and southern China. F) Proportion of cases in northern and southern China each year.

During 1955–2014, a total of 513,034 human brucellosis cases (median 3,504/year [interquartile range (IQR) 1,145–7,886]), including 170 deaths, were reported to the national human brucellosis surveillance system in mainland China (Figure 1, panel A). Among them, 346,682 (67.6%) cases were reported in the individual database during 2004–2014; the proportion of laboratory-confirmed cases ranged from 76.9% in 2004 to 93.2% in 2014 (Table; Figure 2). Most cases during 2004–2014 occurred in males; the male:female ratio was 2.9:1 for both northern (2.9:1) and southern (2.6:1) China (Table; Technical Appendix Table 6). Median age of case-patients was 44 years (IQR 34–54 years), and case distribution was similar by sex and type of diagnosis and between northern and southern China (Figure 2). Most (88.8%) case-patients were farmers or veterinarians or worked in livestock husbandry, transportation, and trade or food production during 2004–2014.

**Table:** Characteristics of persons with brucellosis, mainland China, 2004–2014*

Characteristic	Total, N = 346,682	Northern China, n = 344,204	Southern China, n = 2,478
Type of case			
Confirmed	314,694 (90.8)	312,371 (90.8)	2,323 (93.7)
Probable	31,988 (9.2)	31,833 (9.2)	155 (6.3)
Sex			
M	42,912 (73.8)	42,166 (73.9)	746 (71.0)
F	15,230 (26.2)	14,926 (26.1)	304 (29.0)
Age, y			
Median (IQR)	44.0 (34.1–53.9)	44.0 (34.1–53.9)	46.6 (36.6–56.1)
Age group			
0–4	2,424 (0.7)	2,402 (0.7)	22 (0.9)
5–14	6,638 (1.9)	6,596 (1.9)	42 (1.7)
15–24	25,262 (7.3)	25,108 (7.3)	154 (6.2)
25–34	57,651 (16.6)	57,313 (16.7)	338 (13.6)
35–44	90,777 (26.2)	90,209 (26.2)	568 (22.9)
45–54	87,566 (25.3)	86,901 (25.2)	665 (26.8)
55–64	57,274 (16.5)	56,765 (16.5)	509 (20.5)
>65	19,090 (5.5)	18,910 (5.5)	180 (7.3)
Median delay, d (IQR)			
From illness onset to diagnosis	20.0 (7.5–42.0)	20.0 (7.5–42.0)	20.7 (8.5–47.5)
From diagnosis to report	0.25 (0.04–0.65)	0.25 (0.04–0.65)	0.15 (0.04–0.66)
From illness onset to report	20.6 (7.8–43.4)	20.6 (7.8–43.4)	19.5 (8.0–44.7)
Origin of imported case			
Same county	205,941 (59.4)	204,912 (59.5)	1,029 (41.5)
Other county of same prefecture	92,351 (26.6)	91,628 (26.6)	723 (29.2)
Other prefecture of same province	32,584 (9.4)	32,088 (9.3)	496 (20)
Other province	15,806 (4.6)	15,576 (4.5)	230 (9.3)

*The list of provinces in northern and southern China is available in online Technical Appendix Table 5, https://wwwnc.cdc.gov/EID/article/23/3/16-1710-Techapp1.pdf. Data are presented as no. (%) patients unless otherwise indicated. IQR, interquartile range.

### Overall Incidence and Seasonality

**Figure 3:**
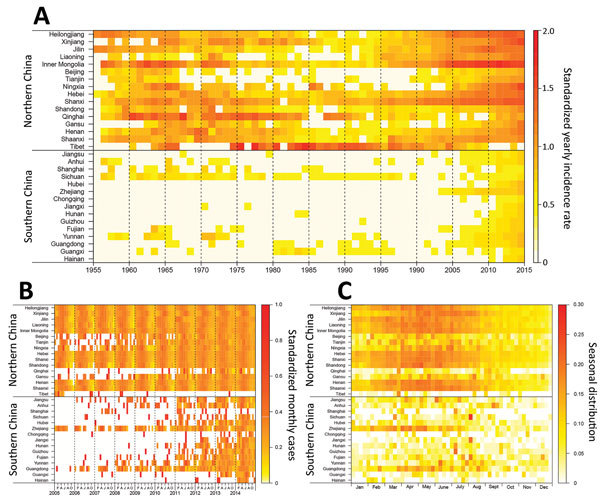
Heat map of provinces with human brucellosis cases, by north and south and the latitude of the capital city of each province, China. A) Time series of incidence rate per 100,000 residents during 1955–2014, standardized by the eighth root. B) Time series of monthly cases, 2005–2014, standardized by the annual number of cases reported by each province. C) Seasonal distribution of cases by province, plotted as the mean value of the proportion of cases in each week of the year from 2005 through 2014.

The annual incidence rate fluctuated during the 60 years studied (Figure 1, panel A). Before 1979, human brucellosis incidence was relatively steady (IQR 0.4–1.0 cases/100,000 residents) and peaked during 1957–1963 (range 0.9–1.8/100,000) and again during 1969–1971 (range 1.0–1.2/100,000). Incidence decreased dramatically beginning in 1979 and remained low until 1994 (IQR 0.05–0.10/100,000). However, the incidence increased from 1995 through 2014 (median 0.2/100,000 [IQR 0.1–0.2] during 1995–2003 and 2.5/100,000 [IQR 1.5–2.9] during 2004–2014); incidence was highest (4.2 cases/100,000 residents) in 2014. After removal of the excess proportion (22.1%; Technical Appendix Figure 1) to account for the potential effect of improvements in data reporting since 2004, the adjusted incidence rate still indicated an average annual growth rate of 20.8% during 2003–2014, and it appears that brucellosis incidence will continue to rise during the next 5 years (Figure 1, panel B). Additionally, 69.1% (258,462/374,141) of the cases were reported in February–July during 1990–2014, with an obvious seasonality in northern China in 2005–2014 (Figure 3).

### Geographic Distribution

Most (99.3%) cases were reported in northern China during 1955–2014, and most provinces in northern China experienced a serious epidemic during the 1950s through the 1970s. Incidence subsequently decreased from the late 1970s through the early 1990s and the disease reemerged in the mid-1990s with cases reported almost every year (Figure 3; Technical Appendix Figure 2). The 5 provinces with the highest median incidence rates during 1955–1994 were Tibet (14.07 cases/100,000 residents), Qinghai (4.43), Shanxi (0.87), Xinjiang (0.35), and Inner Mongolia (0.35); during 1995–2014, highest incidences shifted to Inner Mongolia (25.80), Shanxi (7.33), Heilongjiang (6.07), Jilin (1.79), and Hebei (1.40).

**Figure 4:**
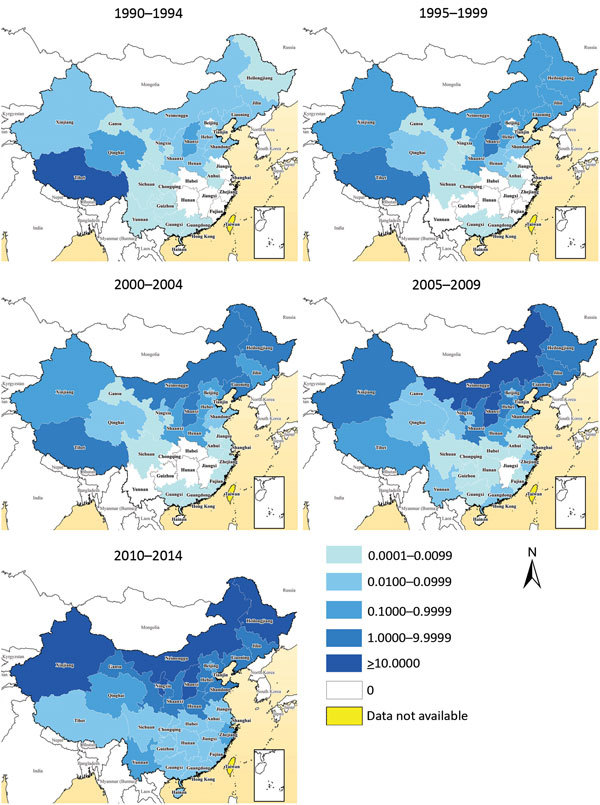
Geographic distribution of the annual incidence rate per 100,000 residents of human brucellosis by 5-year periods, mainland China, 1990–2014.

Similarly, the incidence in southern China has increased since 2000, and human brucellosis has emerged or reemerged in all provinces of southern China. Cases have been reported in almost every province and year since 2010. This finding contrasts with the distribution of brucellosis before 2000, when the disease was limited to a few provinces in southern China, such as Sichuan, Guangxi, and Guangdong (Figures 3, 4; Technical Appendix Figure 2). Additionally, the proportion of imported cases was higher in southern than northern China (58.5% vs. 40.5%; p<0.001), but cases in southern China had a longer lag from illness onset to diagnosis than did those in northern China (21 vs. 20 days; p = 0.003) (Table).

**Figure 5:**
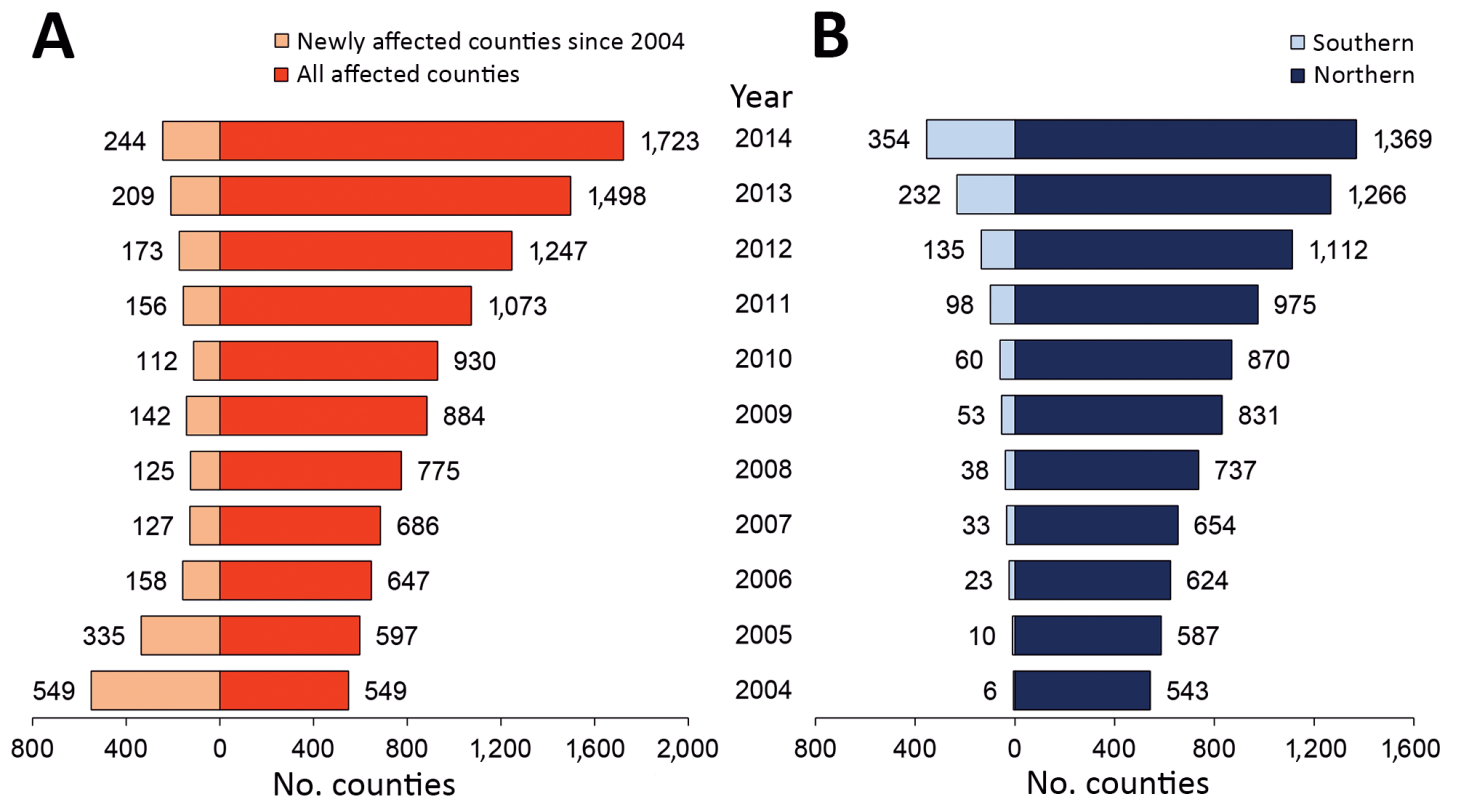
Number of counties with human brucellosis cases, mainland China, 2004–2014. A) Total number of affected counties each year and number of newly affected counties since 2004. B) Total number of affected counties each year in northern and southern China.

**Figure 6:**
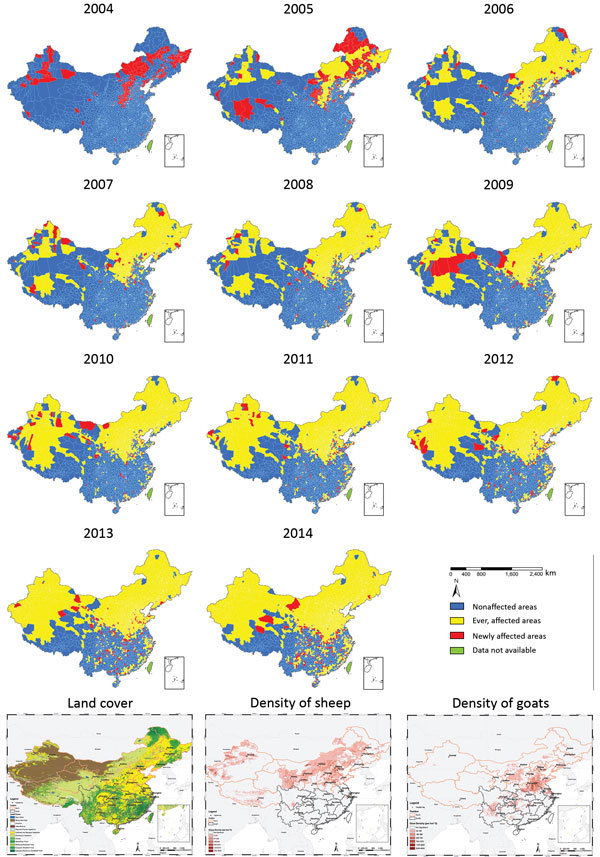
Geographic expansion of human brucellosis across counties and distribution of land covers (*28*) and density of sheep and goats (*29*), mainland China, 2004–2014.

Correspondingly, the number of counties reporting human cases in mainland China increased from 87 in 1993 to 1,723 in 2014; each year since 2004, hundreds of counties were newly affected (Figures 5, 6). The proportion of counties affected in southern China increased from 1.1% in 2004 to 20.5% in 2014, highlighting the spatial spread over the past decade. From a land cover perspective, during 2004–2014, affected areas seem to have expanded from the provinces in northern pastureland areas to the adjacent grassland and agricultural areas that have a high density of sheep and goats, then to coastal areas and southeastern China (Figure 6).

## Discussion

We used a longitudinal surveillance dataset spanning 60 years in China to investigate changes in the epidemiologic characteristics of human brucellosis, especially during the period of dramatic socioeconomic changes during the past 3 decades, during which the urban population increased from 19% in 1980 to 54% in 2014 (*30*). Human brucellosis has reemerged in mainland China since the mid-1990s; incidence has increased and the disease has expanded geographically from northern to southern China. Our study, with long-term and high-quality incidence data, complements a previously published map of human brucellosis in East Asia (*2*–*4*).

The temporal trend in human brucellosis incidence in this study (i.e., high incidence during 1955–1978, low incidence during 1979–1994, and high (and increasing) incidence from 1995 onwards) is consistent with the trend in *Brucella* spp. seroprevalance from animal and human serosurveys conducted in China during 1950–2014. An overall seroprevalence of 41.27% in cattle, sheep, and pigs in brucellosis-endemic areas and 8.43% in humans was reported for 1952–1981, but seroprevalence was only 0.55% in animals and 0.75% in humans during1982–1990 after implementation of a national control program in 1979 (*14*,*31*). During 1990–2001, seroprevalence in humans was 3.28% and has increased steadily since 1995, despite the lack of obvious change in domestic animals (0.36%) (*16*). In the 21st century, seroprevalence maintained an increasing trend in occupationally exposed populations (11.36% during 2001–2004, 13.31% during 2005–2006, 21.97% in 2007, and 22.75% in 2011) and livestock (0.28% during 2001–2004, 0.72% during 2005–2006, and 1.49% in sheep in 2009) (*32*–*35*). Although incidence appeared to increase in every province, Tibet seems to follow an inverse pattern and reported few cases during the past 10 years, but serologic surveys indicate that seroprevalence of *Brucella* infection in animals and humans remained high in some areas since 2005 (*36*). Additionally, this study found a low case-fatality rate in passive surveillance, and the poor follow-up for outcomes of human brucellosis with chronic infection and illness might have contributed.

After brucellosis reemerged in China, the geographic distribution of affected areas gradually expanded (*14*). The areas of brucellosis endemicity gradually shifted from pasturing areas (i.e., Inner Mongolia, Xinjiang, Tibet, Qinghai, and Ningxia) to grassland and agricultural areas (i.e., Shanxi, Liaoning, Hebei, Shandong, and Jilin Provinces), and the southern provinces became increasingly affected (Figures 4, 6) (*14*,*17*). This reemergence and the geographic expansion might be attributed to a variety of contributing factors.

Because brucellosis is not transmitted among humans, humans can be a sentinel for livestock brucellosis. For every human brucellosis case, 15 *B. melitensis* cases are expectedin small ruminants or 150 *B. abortus* cases in cattle (*37*), and the large population of reservoir animals infected with *Brucella* spp. provides a source and is likely to be one of the main causes of infection for humans. The number of livestock dramatically increased during the past 3 decades to meet the growing demand for meat in China (e.g., the yearly numbers of cattle for meat production increased from 3.3 million in 1980 to 46.7 million in 2011, and numbers of sheep and goats increased 6-fold) (*38*), which would have resulted in an increase of the total population of infected animals, even with low-level constant seroprevalence in livestock. The spatial distribution of human brucellosis apparently overlaps with livestock density, especially high densities of sheep and goats (Figure 6), and high incidences of human brucellosis tended to occur most commonly in grasslands at moderate elevation, where sheep and goats are the predominant livestock (*17*,*39*). Another possible reason for the reemergence is the lack of vaccination, quarantine, and elimination of infected animals among backyard livestock. Moreover, the intensive modes of production, which accounted for the rearing of only 42.9% of cattle and 51.1% of sheep and goats in 2011, along with poor infrastructure and lack of high-standard and standardized protocols for maintaining good hygiene within the production cycle, might also result in increasing infections (*38*). Hence persons engaged in livestock husbandry, production, and trade are at high risk for brucellosis infection because of occupational exposure. Additionally, animal products supplied from brucellosis-endemic areas that have not undergone quarantine or pasteurization might increase the risk for infection in nonoccupational populations and urban settings, taking a longer time from illness to diagnosis for imported cases in southern China, which might create extra challenges for disease prevention and case management (*19*,*20*,*40*). Therefore, susceptible livestock animals as the host and infection source for human infections are key to brucellosis prevention and control.

Vaccination is an effective method to reduce brucellosis incidence in livestock and correlates to a decrease in reported human cases, although no vaccines are available for humans (*37*,*41*). Compared with those from other countries, new sequence types of *Brucella* strains have been found in China, and the predominant biovars and sequence types of *Brucella* strains has changed during past half century in some regions (*42*). Thus, new livestock vaccines different from those recommended by the International Office of Epizootics are needed in China.

Targets have been set for brucellosis control in animals in 2015 and 2020 to reach the standard of control and decontamination by province (Technical Appendix Table 7) (*25*). However, to achieve the targets for brucellosis reduction, improvements are needed in socioeconomic parameters, diagnostic and notification systems in animals and humans, and the high prioritization for eliminating the disease in livestock. The continuing existence of human (and animal) brucellosis in China, with potential for further increases in incidence, indicates that the control of brucellosis will not be an easy task without taking a One Health approach, integrating health professionals from the human and animal sectors and administrations. This effort extends beyond medical and veterinary duties and encompasses economic and even political factors (*2*).

Our study has some limitations. First, the data used were collected from passive public health surveillance that might be influenced by changes in surveillance protocols, such as modifications in case definitions and laboratory tests, reporting methods, and availability of health facilities and laboratory diagnostics over the years (Technical Appendix Table 4). Second, individual case data were not reported before 2004, so demographic characteristics, laboratory confirmation, and case distribution could be analyzed only for 2004–2014. Third, data on *Brucella* strains and biotypes and on the varied clinical presentations, including asymptomatic brucellosis infections among humans, were unavailable in this study to explore the distribution of pathogens and the severity of disease. However, the data we used were the most nationally comprehensive for human brucellosis in China.

In view of the reemergence of brucellosis in mainland China and the high incidence, further studies should be conducted to explore the drivers of this situation during the past 2 decades. Livestock–human seroprevalence surveys are needed to understand the correlation between livestock and human brucellosis, to identify the most important animal host species, and to attempt to regress human seroprevalence to livestock prevalence or simply livestock numbers (*43*). The application of spatial–temporal transmission modeling, linking environmental and socioeconomic variables and density (e.g., http://www.worldpop.org) and mobility of livestock and humans (*44*) with the seroprevalence data, would improve understanding of the factors driving reemergence of brucellosis and enable us to better predict the risk in space and time. This information could further inform on potential causes of reemergence (*45*,*46*) and the economics of control in relation to ongoing control activities in China.

In summary, on the basis of notifiable surveillance data in mainland China during 1955–2014, we found that human brucellosis has reemerged since the mid-1990s, and the affected areas have expanded from northern to southern China, especially since 2004. Control strategies in animals and humans should be adjusted to account for these changes by adopting a One Health approach at different levels. Further research is warranted to explore the drivers behind the reemergence.

Technical Appendix Summary of laws or regulations related to brucellosis surveillance and control, mainland China; variables in the aggregated and individual datasets of cases; diagnosis criteria and classification for human brucellosis; geography of each province; demographic and epidemiologic characteristics; standard of control; prediction value for the number of cases in 2004; and heat map of cases, by province.
