# Supplementary material for: Changing Epidemiology of Human Brucellosis, China, 1955–2014
Source: Emerg Infect Dis. 2017 Feb;23(2):184–94. doi: 10.3201/eid2302.151710 (PMC5324817; doi:10.3201/eid2302.151710)
Supplement: Technical Appendix — Summary of laws or regulations related to brucellosis surveillance and control, mainland China; variables in the aggregated and individual datasets of cases; diagnosis criteria and classification for human brucellosis; geography of each province; demographic and epidemiologic characteristics; standard of control; prediction value for the number of cases in 2004; and heat map of cases, by province. [file 15-1710-Techapp-s1.pdf]

Article DOI: http://dx.doi.org/10.3201/eid2302.151710

Changing Epidemiology of Human Brucellosis, China, 1955–2014

Technical Appendix

Technical Appendix Table 1. Summary of laws or regulations related to brucellosis surveillance and control, China, 1955–2014*

No	Title	Approved by	Issued by	Period enforced	Note
1	The Administrative Measures of Infectious Disease	The State Council, PRC, on 1955 Jun 1	The Ministry of Health, PRC on 1955 Jul 5	1955 Jul 5–1978 Sep 19	Undulant fever (brucellosis) was 1 of the 18 notifiable infectious diseases
2	The National Programme of Agricultural Development (1956–1967)	The National People’s Congress, PRC, on 1960 Apr 10 (proposed on 1956 Jan 23)	The National People’s Congress, PRC on 1960 Apr 10	1956–1967	Brucellosis was 1 of diseases for control and elimination, targeted by the Leading Group of Endemic Diseases Prevention and Control in Northern, the CPC Central Committee, established in 1960
3	The Administrative Regulation of Acute Infectious Disease	The State Council, PRC	The Ministry of Health, PRC on 1978 Sep 20	1978 Sep 20	Brucellosis was 1 of the 25 notifiable infectious diseases
4	The Tentative Measures of Brucellosis Control and Prevention	The State Council, PRC on 1979 Dec 22	The Ministry of Health and the Ministry of Agriculture, PRC, on 1980 Jan 31	1980 Mar 1–current	
5	The Law on Infectious Disease Control and Prevention	The Standing Committee of the National People's Congress, PRC, on 1989 Feb 21 and revised on 2004 Aug 28		1989 Sep 1–2004 Nov 30; the revised version during 2004 Dec 1–current	Brucellosis was 1 of the 35 notifiable infectious diseases and listed in Category B (the revised version comprised 37 notifiable diseases)

6	The Implementing Measures of the Law on Prevention and Control of Infectious Diseases	The State Council, PRC, on 1991 Oct 4	The Ministry of Health, PRC, on 1991 Dec 6	1991 Dec 6–current	Brucellosis was listed as the Category B of pathogens for storage, carrying and transport
7	The Regulation on Disease Prevention and Control of Livestock and Poultry	The State Council, PRC, on 1985 Feb 14	The State Council, PRC, on 1985 Jul 1	1985 Jul 1–1997 Dec 31	Brucellosis was 1 of the Category B animal diseases
8	The Implementing Measures of the Regulation on Disease Prevention and Control of Livestock and Poultry	The Ministry of Agriculture, PRC, on 1985 Aug 7; the revised version approved by the 6th Standing Meeting of the Ministry of Agriculture, PRC, in 1991	The Ministry of Agriculture, PRC, on 1985 Aug 7 and revised version issued on 1992 Apr 8	1985 Aug 7–1992 Apr 7; the revised version during 1997 Apr 8–Dec 31	
9	The Law on Disease Prevention and Control of Livestock and Poultry	The Standing Committee of the National People's Congress, PRC, on 1997 Jul 3 and revised on 2007 Aug 30		1998 Jan 1–2007 Dec 31; the revised version during 2008 Jan 1–current	
10	The National Mid- and Long-term Plan of Animal Disease Control and Prevention, 2012–2020	The General Office of State Council, PRC, on 2012 May 20			Brucellosis was 1 of 16 prior domestic animal diseases for control

*CPC, Communist Party of China; PRC, People's Republic of China.

Technical Appendix Table 2. Variables in the aggregated dataset of human brucellosis cases, by year, China, 1955–2003

Variables*	Aggregated	Period
Total cases and no. fatalities	By month at national level	1955–2003
	By province	1955–2003
	By province and month	1980–2003
	By patient sex	1988–1997
		1999–2003
	By age group (each year <10 years of age, each 5-year from 10 to 85 years, and ≥85 years)	1988–1997
		1999–2003
	By occupation	1992–2003
Incidence rate and death rate (per 100,000 residents)	At national level	1955–2003
	By province	1955–2003
The case-fatality rate	At national level	1955–2003
	By province	1980–2003
No. counties with cases	At national level	1994–2003
No. cases imported from other province	At national level	1997–2003

*The data were aggregated and reported monthly by each province in mainland China, 1955 – 2003.

Technical Appendix Table 3. Variables in the individual dataset of human brucellosis cases, mainland China, 2004–2014

Variable*	Definition/classification	Completeness
Identification	A unique 8-digital number for each case.	100% reported
Sex	Male and female	100% reported
Age	Interval from the date of birth to date of onset	100% reported
Zone code of address	Unique 6-digital number at county level	100% reported
Indigenous or imported case	1. Reported by the same county	100% reported
	2. Imported from other county in the same prefecture	
	3. Imported from other prefecture in the same province	
	4. Imported from other province	
Nationality	Chinese or foreigner	100% reported
Occupation	Occupation/status of case-patients	99.5% reported
Type of diagnosis	Probable case (clinical diagnosed case)	100% reported
	Confirmed case (laboratory-confirmed case)	
Date of onset	Date of illness onset	100% reported
Date of diagnosis	Date of diagnosis as a probable or confirmed case	100% reported
Date of report	First date of reporting to dengue surveillance system	100% reported
Date of death	Date of case-patient death, if applicable.	100% reported

*The data were reported by doctors within 24 hours after diagnosis to the online national Notifiable Infectious Disease Reporting Information System (NIDRIS) since 2004. NIDRIS enables all the healthcare institutes across the country to report individual cases of human brucellosis rapidly through the Internet to the data center located in the Chinese Center for Disease Control and Prevention.

Technical Appendix Table 4. Summary of diagnosis criteria and classification for human brucellosis, mainland China*

Variable	1977 Sep–1988 Sep	1988 Oct–1996 Jun	1996 Jul–2007 Oct	2007 Oct–current
Criteria or guidelines	• Tentative Criteria of Diagnosis and Treatment for Human Brucellosis<br>• Tentative Rule of Criteria of Control Zone for Brucellosis and Evaluation Methods at County Level	• Criteria of Diagnosis, Epidemic Area and Control Zone for Brucellosis<br>• Tentative Guidelines for National Brucellosis Sentinel Surveillance	• Diagnostic Criteria and Principles of Management for Human Brucellosis (GB 15988-1995)<br>• Surveillance Standard for Brucellosis (GB16885-1997)<br>• Guidelines for Human Brucellosis Surveillance (2005)	• Diagnostic Criteria for Brucellosis (WS 269-2007)<br>• Guidelines for Diagnosis and Treatment of Human Brucellosis (2012)
Issued by	The Leading Group Office of Endemic Diseases Prevention and Control in Northern (1960–1986), the CPC Central Committee, PRC	The Ministry of Agriculture and the Ministry of Health, PRC	The State Bureau of Technical Supervision, and the Ministry of Health, PRC	The Ministry of Health, PRC
Date issued	1977 Sep and 1981, respectively	1988 Oct 25 and 1990, respectively	1996 Jan 23, 1997 Jun 16, and 2005 Jul 26, respectively	2007 Apr 17 and 2012 Oct 8, respectively

Variable	1977 Sep–1988 Sep	1988 Oct–1996 Jun	1996 Jul–2007 Oct	2007 Oct–current
Date enforced	1977 Sep and 1981, respectively	25 October 1988 and 1990 respectively	1996 Jul 1, 1998 Jan 1, and 2005 Jul 26, respectively	2007 Oct 15 and 2012 Oct 8, respectively
Compared to previous criteria or guidelines	This the first criteria for human brucellosis issued in China	An updated version of previous criteria (left): revised the diagnosis and added the diagnosis criteria of brucellosis in animals	National standards based on previous criteria (left), including the specifications of the laboratory tests for human brucellosis	Based on previous criteria (left), the new national standard and guideline improve the diagnosis and case classification and laboratory tests
Epidemiologic linkage	1.1 Before the onset of illness, the case-patient had a history of close contact with suspected/confirmed animal cases, contaminated animal products, or cultures of *Brucella* spp.; or living in brucellosis-endemic areas; or closely connected with the produce, use, and research of vaccine of *Brucella* spp.			
Clinical description	2.1 Present with undulant, continued, intermittent, or irregular fever (including low fever) of variable duration (some days or weeks), with profuse sweating, fatigue, muscle pain, arthralgia, etc. Lympademia, splenomegaly, and hepatomegaly, Orchitis and epididymitis are common, but small numbers of patients may have jaundice and a variety of rashes; osteoarticular complications are common in patients at chronic phase.			
Laboratory tests	3.1 Presumptive diagnosis<br>• Positive results of plate agglutination test or Bengal plate agglutination test: 0.03 mL serum (++) (criteria used since 1988); or 0.02 mL serum (++) (criteria used during September 1977- September 1988).<br>• Intradermal allergic reaction test by 0.1 mL brucellin: skin redness and infiltration range ≥2.0 cm × 2.0 cm or 4.0 cm^2 observed at 24 h or 48 h (since July 1996); or ≥2.5 cm × 2.5 cm or 6.25 cm^2 observed at 24 h or 48 h (during October 1988–June 1996); or ≥2.5 cm × 2.5 cm observed at 48 h (during September 1977–September 1988).<br>3.2 Serologic diagnosis<br>• Standard tube agglutination test: ≥1:100^{++} in titer; or ≥1:50^{++} in titer for the patient with course over 1 year (since October 2007); or ≥4-fold rise in titer in the paired serum samples after 2–4 weeks of the first test ≥1:100^{++} in titer of the patients with a history of *Brucella* vaccination within half year (since July 1996).<br>• Complement fixation test: ≥1:10^{++} in titer.<br>• Coomb's test: ≥1:400^{++} in titer (since October 1988); ≥1:200^{++} in titer (during September 1977–September 1988).<br>• Cysteine test: ≥1:25^{++} in titer (used only during September 1977–September 1988).<br>3.3 Isolation of *Brucella* spp. from blood or other clinical specimen.			
Diagnosis and classification	4.1 Probable case: a patient with item 1.1 and 2.1.<br>4.2 Confirmed case: a probable case with at least 1 positive result of item 3.1–3.3.	4.1 Probable case: a patient with item 1.1, 2.1, and 3.1.<br>4.2 Confirmed case: a probable case with at least 1 positive result of item 3.2 or 3.3.	4.1 Probable case: a patient with item 1.1, 2.1, and 3.1.<br>4.2 Confirmed case: a probable case with at least 1 positive result of item 3.2 or 3.3.	4.1 Suspected case: a patient with item 1.1 and 2.1.<br>4.2 Probable case: a suspected case with at least 1 positive result of item 3.1.<br>4.3 Confirmed case: a suspected or probable case with at least 1 positive result of item 3.2

Variable	1977 Sep–1988 Sep	1988 Oct–1996 Jun	1996 Jul–2007 Oct	2007 Oct–current
				or 3.3.<br>4.4 Latent infection: a person with item 1.1 and at least 1 positive result of item 3.2 or 3.3, but without item 2.1.

*There were not criteria/guidelines for human brucellosis diagnosis issued by the Chinese national health authorities during 1950–1976, but human brucellosis cases are diagnosed by clinical manifestations, epidemiologic links, and/or available laboratory tests at that time including intradermal allergic reaction test, agglutination test and culture, and so on. CPC, Communist Party of China; GB, Guo-Biao (National Standard); PRC, People's Republic of China; WS, Wei-Sheng (Standard in Public Health).

Technical Appendix Table 5. Summary of the geography of each province in mainland China

No.	Province	Zone code	Climate*	Inland or coastal province	Northern or southern*	Adjacent country	Capital city	Latitude†	Longitude
1	Heilongjiang	230000	Mid-temperate	Inland	Northern	Russia	Harbin	46.1138	126.185
2	Jilin	220000	Mid-temperate	Inland	Northern	Russia and North Korea	Changchun	44.1156	125.352
3	Xinjiang	650000	Mid-temperate	Inland	Northern	Russia, Mongolia, Kazakhstan, Kyrgyzstan, Tajikistan, Afghanistan, Pakistan, and India	Urumqi	43.7878	87.574
4	Inner Mongolia	150000	Mid-temperate	Inland	Northern	Russia and Mongolia	Hohhot	40.7632	110.82
5	Liaoning	210000	Warm-temperate	Coastal	Northern	North Korea	Shenyang	40.6843	122.589
6	Beijing	110000	Warm-temperate	Inland	Northern	None	Beijing	39.94	116.41
7	Tianjin	120000	Warm-temperate	Coastal	Northern	None	Tianjin	39.16	117.2
8	Hebei	130000	Warm-temperate	Coastal	Northern	None	Shijiazhuang	38.1269	115.078
9	Shanxi	140000	Warm-temperate	Inland	Northern	None	Taiyuan	37.8098	112.8
10	Ningxia	640000	Mid-temperate	Inland	Northern	None	Yinchuan	37.6234	106.026
11	Qinghai	630000	Cold	Inland	Northern	None	Xining	36.6401	101.835
12	Shandong	370000	Warm-temperate	Coastal	Northern	None	Jinan	36.313	118.368
13	Gansu	620000	Mid-temperate	Inland	Northern	Mongolia	Lanzhou	35.5751	104.657
14	Henan	410000	Warm-temperate	Inland	Northern	None	Zhengzhou	34.707	113.058
15	Shaanxi	610000	Warm-temperate	Inland	Northern	None	Xi'an	34.3038	108.849
16	Tibet	540000	Cold	Inland	Northern	India, Bhutan, Nepal, Myanmar, and Pakistan	Lhasa	29.65	91.13
17	Jiangsu	320000	Subtropical	Coastal	Southern	None	Nanjing	32.8614	118.575
18	Anhui	340000	Subtropical	Inland	Southern	None	Hefei	31.8527	117.543
19	Shanghai	310000	Subtropical	Coastal	Southern	None	Shanghai	31.28	121.46
20	Hubei	420000	Subtropical	Inland	Southern	None	Wuhan	30.8781	112.606
21	Sichuan	510000	Subtropical	Inland	Southern	None	Chengdu	30.2459	103.978
22	Zhejiang	330000	Subtropical	Coastal	Southern	None	Hangzhou	29.9769	120.444

23	Chongqing	500000	Subtropical	Inland	Southern	None	Chongqing	29.59	106.55
24	Jiangxi	360000	Subtropical	Inland	Southern	None	Nanchang	28.2274	115.261
25	Hunan	430000	Subtropical	Inland	Southern	None	Changsha	27.3878	113.006
26	Guizhou	520000	Subtropical	Inland	Southern	None	Guiyang	27.3627	106.816
27	Fujian	350000	Subtropical	Coastal	Southern	None	Fuzhou	25.337	118.827
28	Yunnan	530000	Subtropical	Inland	Southern	Vietnam, Laos, and Myanmar	Kunming	24.8119	103.034
29	Guangdong	440000	Subtropical	Coastal	Southern	None	Guangzhou	22.9286	113.414
30	Guangxi	450000	Subtropical	Coastal	Southern	Vietnam	Nanning	22.85	108.37
31	Hainan	460000	Tropical	Coastal	Southern	None	Haikou	19.5855	110.101

*The general climate of each province, which is available on the website of China Meteorological Administration (http://www.cma.gov.cn/). Each province is categorized as temperate northern province (16 provinces) or subtropical southern province (15 provinces) from previous study (Feng L, et al. Influenza-associated mortality in temperate and subtropical Chinese cities, 2003-2008. Bull World Health Organ. 2012,90:279–88).

†The latitude and longitude of capital city of each province.

Technical Appendix Table 6. Demographic and epidemiologic characteristics of human brucellosis cases, mainland China, 2004–2014*

Characteristic	Total, N = 346,682	Male, n = 258,238	Female, n = 88,444
Type of case			
Confirmed	314,694 (90.8)	233,615 (90.5)	81,079 (91.7)
Probable	31,988 (9.2)	24,623 (9.5)	7,365 (8.3)
Age, y			
Median (IQR)	44.0 (34.1–53.9)	43.9 (34.0–53.7)	45.0 (35.0–54.0)
Age group			
0–4	2,424 (0.7)	1,446 (0.6)	978 (1.1)
5–14	6,638 (1.9)	4,401 (1.7)	2,237 (2.5)
15–24	25,262 (7.3)	19,800 (7.7)	5,462 (6.2)
25–34	57,651 (16.6)	44,407 (17.2)	13,244 (15)
35–44	90,777 (26.2)	68,185 (26.4)	22,592 (25.5)
45–54	87,566 (25.3)	63,824 (24.7)	23,742 (26.8)
55–64	57,274 (16.5)	41,837 (16.2)	15,437 (17.5)
≥65	19,090 (5.5)	14,338 (5.6)	4,752 (5.4)
Year of onset			
2004	11,477 (3.3)	8,818 (3.4)	2,659 (3.0)
2005	18,416 (5.3)	13,972 (5.4)	4,444 (5.0)
2006	19,014 (5.5)	14,359 (5.6)	4,655 (5.3)
2007	19,723 (5.7)	14,773 (5.7)	4,950 (5.6)
2008	27,771 (8.0)	20,672 (8)	7,099 (8.0)
2009	35,824 (10.3)	26,421 (10.2)	9,403 (10.6)
2010	33,786 (9.7)	24,822 (9.6)	8,964 (10.1)
2011	38,183 (11.0)	28,552 (11.1)	9,631 (10.9)
2012	39,607 (11.4)	29,869 (11.6)	9,738 (11.0)
2013	44,739 (12.9)	33,068 (12.8)	11,671 (13.2)
2014	58,142 (16.8)	42,912 (16.6)	15,230 (17.2)
Month of onset			
January	22,315 (6.4)	16,898 (6.5)	5,417 (6.1)
February	26,571 (7.7)	20,585 (8.0)	5,986 (6.8)
March	37,061 (10.7)	28,451 (11.0)	8,610 (9.7)
April	43,402 (12.5)	33,203 (12.9)	10,199 (11.5)
May	49,472 (14.3)	37,488 (14.5)	11,984 (13.5)
June	45,588 (13.1)	33,788 (13.1)	11,800 (13.3)
July	37,827 (10.9)	27,390 (10.6)	10,437 (11.8)
August	27,659 (8.0)	19,840 (7.7)	7,819 (8.8)
September	16,786 (4.8)	11,880 (4.6)	4,906 (5.5)
October	14,677 (4.2)	10,416 (4.0)	4,261 (4.8)
November	13,555 (3.9)	9,738 (3.8)	3,817 (4.3)
December	11,769 (3.4)	8,561 (3.3)	3,208 (3.6)
Median delay, d (IQR)			
From illness onset to diagnosis	20.0 (7.5–42.0)	20.0 (7.7–42.0)	19.8 (6.8–42.6)

Characteristic	Total, N = 346,682	Male, n = 258,238	Female, n = 88,444
From diagnosis to report	0.25 (0.04–0.65)	0.24 (0.04–0.65)	0.25 (0.04–0.65)
From illness onset to report	20.6 (7.8–43.4)	20.7 (8.4–43.3)	20.5 (7.4–43.6)
Where were the cases imported from			
The same county	205,941 (59.4)	153,231 (59.3)	52,710 (59.6)
Other county of same prefecture	92,351 (26.6)	68,584 (26.6)	23,767 (26.9)
Other prefecture of same province	32,584 (9.4)	24,415 (9.5)	8,169 (9.2)
Other province	15,806 (4.6)	12,008 (4.6)	3,798 (4.3)

*Data are presented as no. (%) patients unless otherwise indicated. IQR, interquartile range.

Technical Appendix Table 7. Summary of standard of brucellosis control, mainland China

County-level category	Criteria*
Criteria of control area	Meeting the following 3 requirements at least 2 consecutive years:<br>1. For livestock vaccinated >18 months of age or without vaccination, sampling >3,000 serum samples in pastoral areas, >1,000 serum samples in agricultural areas and semiagricultural and semipastoral areas, and detected by tube agglutination test (seropositive <0.5% for sheep, goats, and deer, <1% for cattle, and <2% for pigs) or complement fixation test (<0.5%).<br>2. Sampling >200 (total) abortion specimens from sheep, goats, cattle, and pigs (if the number of abortions is insufficient, sampling normal placenta, breast milk, vaginal secretions, or spleen), and without *Brucella* spp. detected.<br>3. All animals infected with *Brucella* have been culled with biosafety disposal.
Criteria of stable control area	Meeting the following 3 requirements at least 3 consecutive years using the same requirement of sampling and laboratory tests in the criteria of control area:<br>1. Seropositive <0.1% for sheep or goats, <0.3% for pigs, <0.2% for cattle and deer.<br>2. No *Brucella* detected from specimens of sheep, goats, cattle, and pigs.<br>3. All animals infected with *Brucella* have been culled with biosafety disposal.
Criteria of decontaminated area	Meeting the following 2 requirements at least 2 consecutive years using the same requirement of sampling and laboratory tests in the criteria of control area:<br>1. No outbreak of brucellosis within 2 years after meeting the criteria of stable control area.<br>2. All specimens are negative by tube agglutination test or complement fixation test.

*The Ministry of Agriculture, People's Republic of China. Technical specification of brucellosis control and prevention. 2006 [cited 2015 Aug 28]. http://www.moa.gov.cn/zwllm/nybz/200803/P020080429675112156434.doc

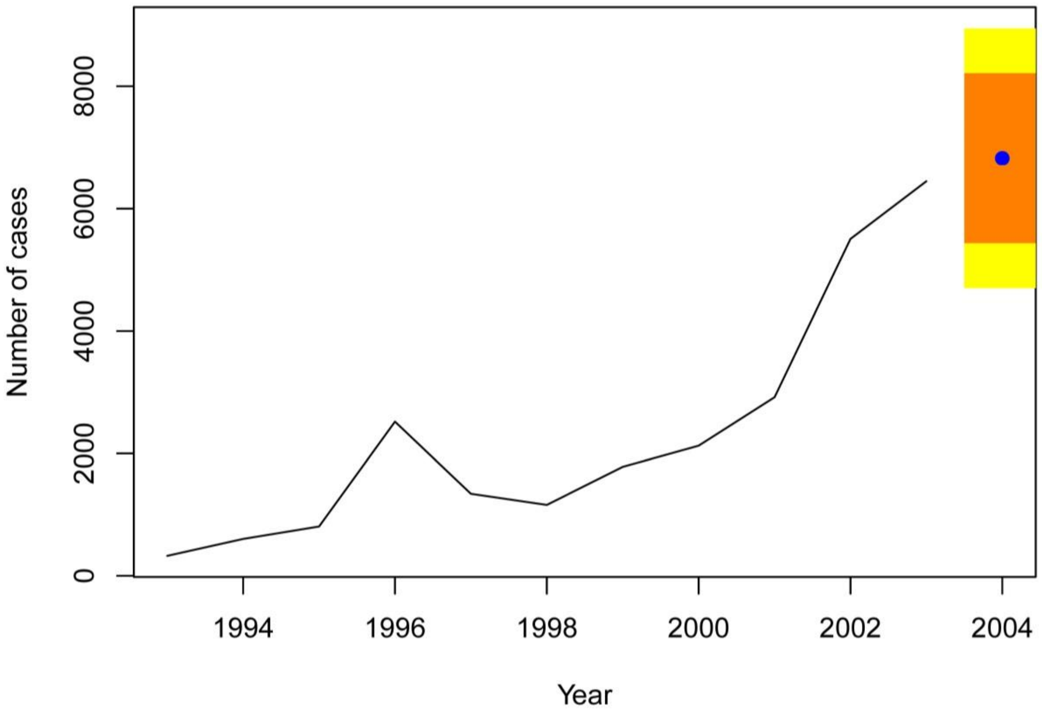

Technical Appendix Figure 1. Prediction value (6,823) for the number of human brucellosis cases in 2004 by the Holt's exponential smoothing method. The 80% CIs (orange) and 95% CIs (yellow) of the prediction value are 5,436–8,211 and 4,701–8,945 cases respectively. Comparing to this upper limited value (8,945) of the 95% CI, the actual number of cases (11,477) in 2004 has an excess proportion of 22.1%.

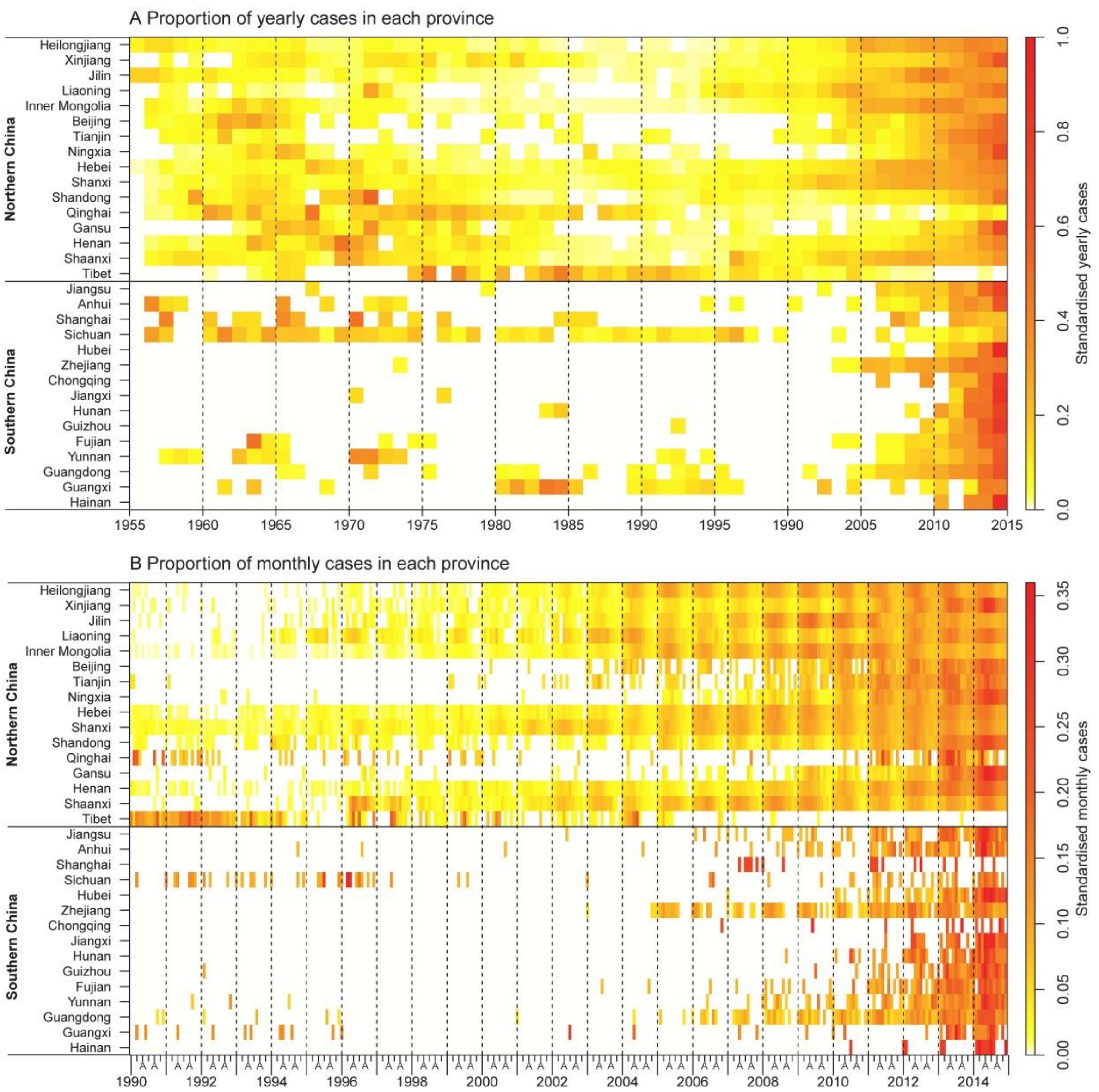

Technical Appendix Figure 2. Heat map of human brucellosis cases, by province, sorted by north and south and the latitude of capital city of each province, mainland China. A) Time series of the annual number of human brucellosis cases by province during 1955–2014, standardized by the total number of cases in each province. B) Time series of the monthly number of human brucellosis cases by province during 1990–2014, standardized by the total number of cases in each province.
